# Three-Dimensional Scanning Virtual Aperture Imaging with Metasurface

**DOI:** 10.3390/s25010280

**Published:** 2025-01-06

**Authors:** Zhan Ou, Yuan Liang, Hua Cai, Guangjian Wang

**Affiliations:** Huawei Technologies Co., Ltd., Chengdu 610000, China; ouz2021@yeah.net (Z.O.); liangyuan22@huawei.com (Y.L.); bruce.caihua@huawei.com (H.C.)

**Keywords:** metasurface, scanning, near field, RMA, DOF

## Abstract

Metasurface-based imaging is attractive due to its low hardware costs and system complexity. However, most of the current metasurface-based imaging systems require stochastic wavefront modulation, complex computational post-processing, and are restricted to 2D imaging. To overcome these limitations, we propose a scanning virtual aperture imaging system. The system first uses a focused beam to achieve near-field focal plane scanning, meanwhile forming a virtual aperture. Secondly, an adapted range migration algorithm (RMA) with a pre-processing step is applied to the virtual aperture to achieve a 3D high-resolution reconstruction. The pre-processing step fully exploits the feature of near-field beamforming that only a time delay is added on the received signal, which introduces ignorable additional calculation time. We build a compact prototype system working at a frequency from 38 to 40 GHz. Both the simulations and the experiments demonstrate that the proposed system can achieve high-quality imaging without complex implementations. Our method can be widely used for single-transceiver coherent systems to significantly improve the imaging depth of field (DOF).

## 1. Introduction

Metasurface-based imaging is emerging as a promising technology in security screening, nondestructive detection, and the remote monitoring of people [1,2,3,4,5]. Compared with conventional radio-frequency imaging systems, metasurface-based imaging possesses the advantages of lower hardware costs and reduced system complexity. The mainstream metasurface-based imaging modality is called coded aperture imaging [6]. Low-correlated illumination patterns are generated to encode the scene information into a few measurements, enabling image reconstruction through computation. Coded aperture imaging usually requires fewer measurements than conventional imaging modality, resulting in a faster imaging speed. However, the modulation pattern design and computational burden still pose challenges.

The simplest method of radiation modulation is to use random illuminations by introducing an extra mask, such as a metallic mask [7] or a spinning disk [8]. Due to its capability to arbitrarily manipulate the wavefront of electromagnetic waves, the metasurface can generate random illuminations more effectively. Frequency-diverse metasurface antennas have been widely used, achieving high imaging resolution and speed [9,10]. Since frequency-diverse metasurface antennas require an ultra-wide working bandwidth, dynamic metasurface antennas [11,12,13], and frequency-polarization sensitive metasurface antennas [14,15] are proposed for more flexible applications. To achieve a high image reconstruction SNR and accuracy, the modulation patterns must be measured carefully to form a precise forward model. A more effective strategy is to use orthogonal pattern sets such as Hadamard basis [16,17,18] or Fourier basis [19]. Despite the elimination of the cumbersome calibration process, these methods still require complex computational reconstruction, such as matched filter or iterative optimization.

Besides the complexity of the modulation pattern design, measurement, and preprocess computation, many metasurface-based imaging methods are only suited for two-dimensional (only cross-range) [20,21,22] but are not straightforward to be extended to three-dimensional. Recently, considerable research has achieved significant progress in 3D reconstruction [23,24,25,26,27]. The common basic idea is to convert the metasurface-based imaging problem into a conventional synthetic aperture radar (SAR) imaging problem. Specifically, a pre-processing step is performed to decompress the raw received signal as if it had been transmitted and received from a set of uniformly distributed antennas. However, there are still some drawbacks inherent in these methods. First, random illumination patterns are adopted, which require careful designing to ensure their orthogonality and completeness. Second, all the random patterns need precise measurements to calibrate a precise forward model. Typically, tens, or even one hundred, measurements are required. This procedure can be very time-consuming since each measurement requires scanning at the whole region of interest (ROI) by an extra receiver. Third, when converting the forward model to apply the SAR algorithm, inverse matrix calculations are performed for each different modulation pattern and frequency, resulting in a huge computation and memory burden. All these drawbacks restrict the applications of the above methods, leading to mere simulations or poor experimental results.

Here, we propose a scanning virtual aperture (SVA) imaging system to overcome the aforementioned limitations. Our system combines the advantages of both a raster-scanning system and a synthetic aperture system, yielding 3D high-resolution imaging results by simple illumination modulation without any complex calibrations or computations. For the illumination, we use a focused beam to scan the scene, leading to a 2D image with a very limited DOF. In other words, the metasurface is used as a focusing lens with a fixed focal length. Noticing that the focal plane composes a new virtual aperture, we multiplex raw acquisitions using digital coherent superposition to provide high resolution at all depths. In order to accelerate the reconstruction speed, we develop an adjusted RMA to reconstruct the 3D image. Benefitting from the unique near-field focusing modulation pattern, there is only one extra phase shift step. Compared with the current 3D imaging method, our system avoids any complex modulation pattern design, calibration, and matrix computation, providing a feasible and effective solution to achieve high-quality reconstruction of the whole 3D scene.

## 2. Methods

**Figure 1:**
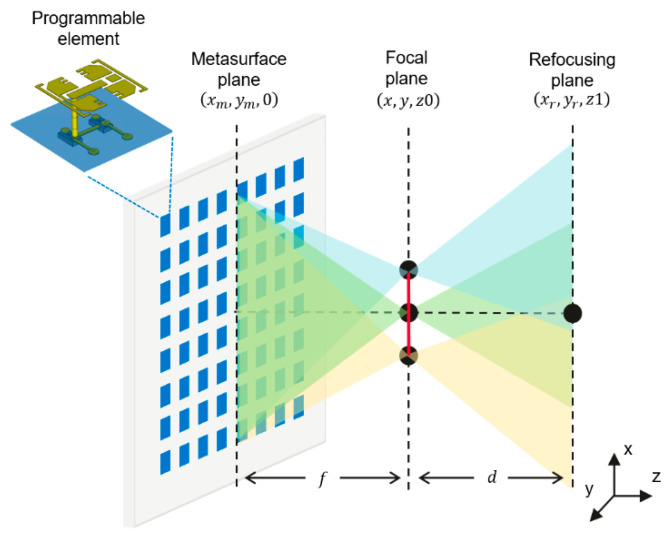
Principle of scanning virtual aperture imaging. The virtual aperture is indicated by the red line.

Figure 1 illustrates the principle of SVA imaging. The metasurface consists of several programmable elements, with agile phase modulation. The desired phase modulation can be generated by tuning the active components on the programmable elements. Based on the scalar diffraction model in wave optics, the echo signal neglecting attenuation can be expressed as:

(1) $$S(\varphi ,k)=∭\rho (x,y,z)\cdot \exp(-j2kr)\cdot \exp(j2\varphi )\cdot \exp(j2\varphi_{0})dxdydz,$$

where k is the wavenumber, S(φ,k) is the echo signal when the modulation phase is φ, and $\varphi_{0}$ is the initial phase caused by the incident angle of the feed horn. This system uses the metasurface as a focusing lens with a fixed focal length f to scan the plane (x,y,z0). Therefore, the modulation phase on the metasurface should be a converging spherical wave, which can be expressed as

(2) $$\begin{array}{cc} \varphi (x,y) & =k_{c}r_{mf}-\varphi_{0}\\ & =k_{c}\sqrt{{\left(x_{m}-x\right)}^{2}+{\left(y_{m}-y\right)}^{2}+f^{2}}-\varphi_{0}, \end{array}$$

where $k_{c}$ is the central wavenumber. This modulation phase converts S(φ,k) into a focused image in the cross-range S(x,y,k). Next, inverse fast Fourier transform (IFFT) is applied to obtain a range-focused image, which can be represented as:

(3) $$\rho (x,y,z){|}_{z0}={\mathrm{FT}}_{k}^{-1}\left\{S(x,y,k)\right\},$$

where ${\mathrm{FT}}_{k}^{-1}$ denotes IFFT along the k dimension. However, this image is only well-focused in the focal plane and is otherwise defocused due to beam diffraction. A straightforward way to get well focused 3D images is to change the focal length to different distances, whereas very time-consuming. Here, we note that the focal plane can be viewed as a new virtual aperture, allowing for the application of a synthetic aperture technique. For the target point $\left(x_{r},y_{r},z1\right)$ far away from the focal plane, it can be digitally refocused by coherent superposition of the echo signals, which can be expressed as:

(4) $$\rho (x,y,z){|}_{z1}={\mathrm{FT}}_{k}^{-1}\left\{\iint S(x,y,k)\cdot \exp(2jk_{c}\sqrt{{\left(x_{r}-x\right)}^{2}+{\left(y_{r}-y\right)}^{2}+d^{2}})dxdy\right\},$$

where d is the distance between the focal plane and the refocusing plane. In fact, (4) is a back projection algorithm. It constructs an identified matched filter for each pixel sequentially, resulting in high complexity. To improve the efficiency, we further develop an adjusted RMA. Our method only requires an extra phase shift to adapt the RMA, which can be expressed as:

(5) $$S^{VA}(x,y,k)=S(x,y,k)\cdot \exp(-2jkf),$$

where $S^{VA}$ is the signal on the virtual aperture. According to the Fourier relationship between time and frequency, (5) adds a temporal delay to the captured signal. Hence, all the waves seem to be emanating from the focal plane. Ignoring the different time delays at different scanning points due to the relatively small scanning area, this pre-processing step corrects the mismatch between the wavefront and the conventional imaging model. Therefore, we can use RMA to reconstruct the 3D image with spatially invariant resolution, which is only dependent on the size of the metasurface. Due to the page limitations, we do not review RMA in detail here [28,29], but directly provide the reconstruction formula as:

(6) $$\rho (x,y,z){|}_{z>f}={\mathrm{FT}}_{3D}^{-1}\left\{{\mathrm{Interp}}_{k\to k_{z}}\left[S^{VA}(x,y,k)\right]\right\},$$

where $k_{z}$ is the projection of the spatial wave vector on the axial direction, and Interp is the Stolt interpolation. Finally, as shown in Figure 2, the 3D reconstruction result can be composited by the scanning image (3) and the RMA reconstruction (6):

(7) $$\rho (x,y,z)=\left\{\begin{array}{l} {\mathrm{FT}}_{k}^{-1}\left\{S(x,y,k)\right\},\;z=f\\ {\mathrm{FT}}_{3D}^{-1}\left\{{\mathrm{Interp}}_{k\to Q_{z}}\left[S^{VA}(x,y,k)\right]\right\},\;z>f. \end{array}\right.$$

The system’s lateral resolution is dependent on the real aperture size of the metasurface, which is given by:

(8) $$\delta_{x}=\frac{\lambda_{c}}{4sin\left(\theta /2\right)},$$

where $\lambda_{c}$ is the central wavelength, and θ is the angle subtended by the metasurface aperture. The axial resolution is given by:

(9) $$\delta_{z}=\;c/2B,$$

where c is the light speed, and B is the bandwidth of the signal.

## 3. Results

### 3.1. Simulation

First, a simulation is conducted to evaluate the performance of the proposed algorithm. The parameters except the working frequency are slightly different from those of the experimental prototype system for better presentation. The linear frequency sweep from 38 GHz to 40 GHz, with 31 sampling points, is applied in the simulation. The bandwidth of 2 GHz leads to the range resolution of 7.5 cm. The metasurface has 51 × 51 elements, with $\lambda_{c}/2$ spacing. The imaging FOV and scanning area are both 25 × 25 cm^2^. The resolution step size and sampling interval are both set to 5 mm. The focal length is set as 30 cm. The scene consists of three points at (0, −6, 30), (0, 0, 50), and (0, 6, 90) cm. The initial phase and quantization of the metasurface are ignored.

**Figure 3:**
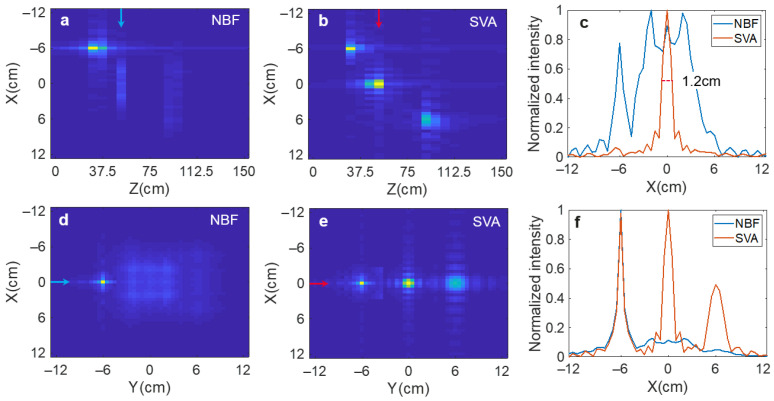
Simulation results of the comparison between NBF and SVA. (**a**,**b**) Axial plane of the targets. (**d**,**e**) Lateral plane of the maximum intensity projection. (**c**) Intensity profiles along the line indicated by the arrow in the axial plane. (**f**) Intensity profiles along the line indicated by the arrow in the lateral plane.

Figure 3 shows the simulation results using near-field beamforming (NBF) and SVA with the same illumination modulation. While NBF only focuses the target in the focal plane, SVA satisfactorily reconstructs all targets at different distances. The DOF of NBF is defined as twice the Rayleigh length [30], which can be calculated as:

(10) $$\Delta L_{c}=\pi \delta_{x}^{2}/\lambda =\frac{\pi \lambda }{16\;\sin^{2}(\theta /2)}=1.62\;\mathrm{cm},$$

which is very limited. As a result, these targets not in the focal plane are all defocused. SVA obviously improves the DOF, leading to high resolution at all distances (Figure 3f). We take the middle target for resolution analysis quantitatively. The full-width at half-maxima (FWHM) is 1.2 cm (Figure 3c) with little deviation from the theoretical value $\delta_{x}=1\;\mathrm{cm}$ according to (8).

**Figure 4:**
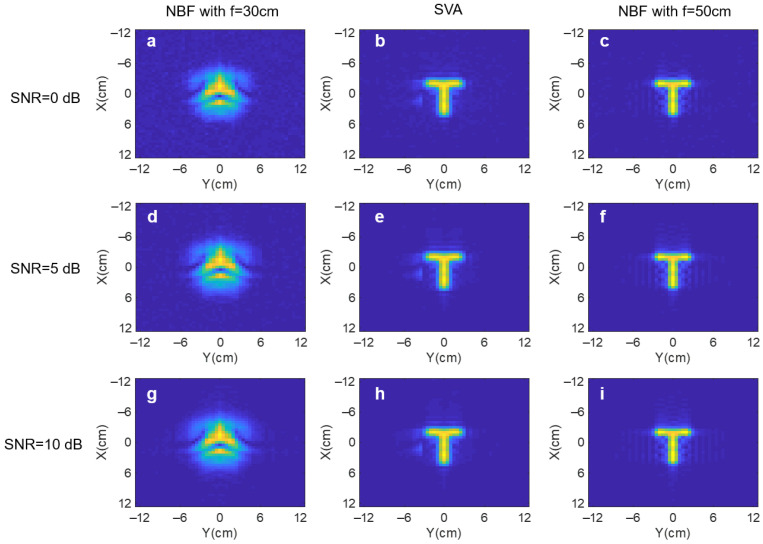
Simulation results with different SNRs. Top row: SNR = 0 dB. Mid row: SNR = 5 dB. Bottom row: SNR = 10 dB. (**a**,**d**,**g**) Defocused NBF. (**b**,**e**,**h**) SVA. (**c**,**f**,**i**) In-focus NBF.

Next, we evaluate the performance with different SNRs. The parameters are kept the same with channel SNR set as 0 dB, 5 dB, and 10 dB. The target is a letter “T” at the distance of 50 cm. As shown in Figure 4, defocused NBF fails to recover the shape. However, SVA successfully recovers the “T” shape with any SNR. The noise mainly generates artifacts in the background with little impact on the target contour. We further compare the SVA with the in-focus NBF results, where the focal length is set as equivalent to the focal length of 50 cm. The SVA and in-focus NBF results are very similar, as shown in the middle and right columns of Figure 4. Therefore, we demonstrate that the proposed method is very robust to noise with high reconstruction accuracy.

### 3.2. Experiment

**Figure 5:**
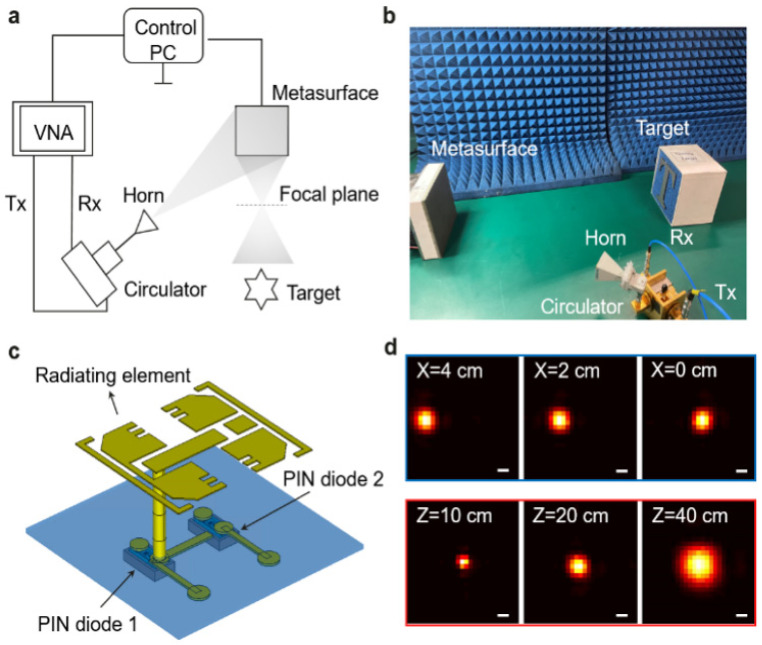
Experimental imaging system. (**a**) Schematic of the metasurface-based imaging system. (**b**) Photograph of the system. (**c**) Main structure of the programmable element. Only the radiating layer and PIN layer are displayed for clarity. (**d**) Near field focusing spot in the lateral plane. Top row: different lateral location with fixed Z = 20 cm. Bottom row: different axial location with fixed X = 0 cm. All the spot sizes are close to the diffraction limit. Scale bar: 1 cm.

The experiment setup is shown in Figure 5. Unlike most experimental prototype systems with separated transmitter and receiver placed side by side, our system introduces a circulator to form a quasi-monostatic configuration. This design is crucial for creating a reversible electromagnetic wave path, leading to a high signal-to-noise ratio (SNR) for confocal scanning. The vector network analyzer (VNA) connected with the transceiver is used to generate linear stepped-frequency signals from 38 GHz to 40 GHz. The metasurface comprises 32 × 32 elements with a whole aperture size 12 × 12 cm^2^. The main structure of each element is shown in Figure 5c. Each radiating element is connected by a driver circuit consisting of two PIN diodes and switches. The circuit is controlled by the field programmable gate array (FPGA) with 12 V applied voltage. By applying a bias signal, we control the resistance and capacitance in the switch to turn the diodes to ON/OFF. Finally, we can obtain four different phase states by using different combinations of the diodes states. These phases are experimentally tested to be 0°, 144°, 294°, and 87°. Due to the high power loss of the last state, we only use the first three phases, i.e., 1.5 bit quantization. When generating the focusing beam, we directly set the closest value to the theoretical value, according to (2). In general, higher quantization accuracy leads to better focus quality. Given only 1.5 bit quantization, whether the beam can be focused as expected is a concern. To validate the focusing quality, we measured the beam focus at 10 different lateral and axial locations. Figure 5d shows three typical beam spots in the lateral plane. All the beam spots are well focused, thus proving that 1.5 bit quantization is sufficient to implement the desired phase. Since our method can be applied to arbitrary quantization, we do not pay special attention to any further optimization. The metasurface is illuminated by a feed horn antenna at about 30° and 25 cm for appropriate beam coverage. The scanning range is the same as the imaging FOV to be 10 × 10 cm^2^. The sampling interval is set to 2.5 mm in each axis thus 41 × 41 points are scanned in total. It takes about 0.2 s for the data reading and writing of the VNA, and 0.3 s for the phase change of the metasurface, adding up to 0.5 s to collect the data for one point. Ultimately, it takes about 15 min to complete an entire scan. The speed can be significantly improved by substituting the VNA with a high-speed RF link and optimizing the control software. Since both the metasurface and the VNA exhibit high stability, there is no extra time cost for system calibration during our experiments.

**Figure 6:**
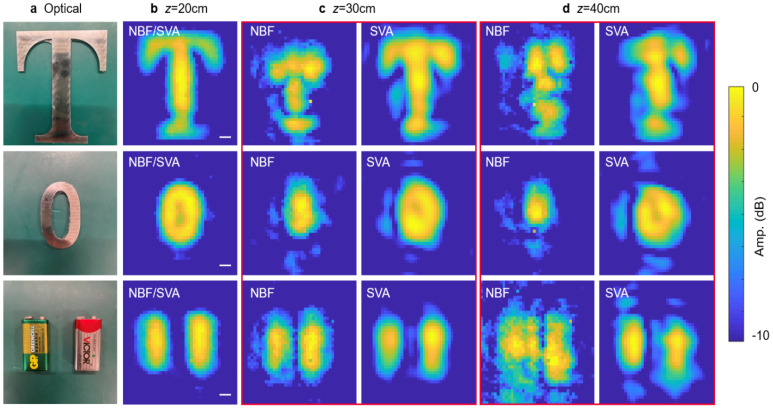
Experimental imaging results of targets at different distances with a fixed focal length. (**a**) Optical images. The distance between the metasurface and the targets is set as (**b**) 20 cm, (**c**) 30 cm, and (**d**) 40 cm. Scale bar: 1 cm.

We validate the 2D imaging capability at different distances. To reduce environmental noises, we sample the background signal first and subtract it from the target signal. The focal length is fixed at 20 cm. We set the targets at 20 cm, 30 cm, and 40 cm, respectively. As shown in Figure 6, our system displays the highest resolution and SNR at the distance of z=20 cm.The main shape along with some fine structures of the targets are well reconstructed. For example, the hole in the metal letter “zero” is just discernible (Figure 6b). Since the lateral size of the hole is 1.5 cm, we can estimate that the resolution of our system is $\delta_{x}<1.5\;\mathrm{cm}$, which is consistent with the measured spot size (Figure 5d). The imaging result of SVA is the same as NBF when z=f, both obtained by direct scanning. For cases of greater distance, the imaging results of NBF are obviously defocused, leading to a low resolution and SNR. However, SVA results remain relatively good. We evaluate the quality of the reconstructed image I(x,y) with the normalized mean squared error (NMSE):

(11) $$\mathrm{NMSE}=\frac{\sum_{x,y}{\left(I(x,y)-I_{\mathrm{ref}}(x,y)\right)}^{2}}{\sum_{x,y}I_{\mathrm{ref}}^{2}(x,y)},$$

where the imaging result at z=20 cm is used as the reference image $I_{\mathrm{ref}}(x,y)$. The results are listed in Table 1. The NMSE value of SVA is much lower than that of NBF, demonstrating better imaging quality. We also compare the computational time between SVA and other typical 3D reconstruction methods, as shown in Table 2. SVA takes 0.001 s for the pre-processing and 0.3 s for RMA on a 64-bit 3 GHz CPU. Due to the different computing platform, we further consider the time ratio between pre-processing and RMA for a fair comparison. This ratio of SVA is only 0.003, showing great superiority over the other methods. In addition, it is emphasized that no extra time is required for measuring and calibrating the modulation patterns before pre-processing, making SVA an effective 3D imaging with metasurface.

**Figure 7:**
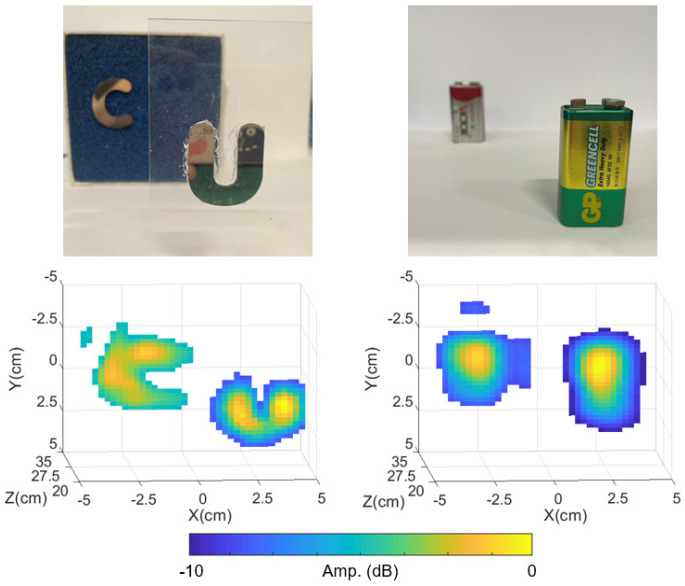
The 3D imaging results of our proposed system.

Finally, we demonstrate the capability of the proposed system for real 3D imaging, while previous studies mainly report results using a single target at a fixed distance. The experiments are conducted with objects at different distances, as shown in Figure 7. The focal length is maintained at 20 cm. For the case letter “UC”, the letter “U” is placed at 20 cm, while the letter “C” is placed at 30 cm. For the two-battery case, the front battery is placed at 25 cm, while the back battery is placed at 35 cm. Our system successfully provides a 3D reconstruction of the scene, revealing the shape and location of each object.

## 4. Discussion

The proposed SVA system exhibits many connections and distinctions with the conventional focal plane scanning (FPS) system and the SAR. Both SVA and FPS employ raster-scanning with a focused beam, but SVA has a larger DOF owing to the application of the synthetic aperture algorithm. On the other hand, SAR creates aperture from the platform motion, whereas there is no motion in SVA. SVA creates aperture with the metasurface, which is acting like a scanning lens. In this regard, SVA is similar to interferometric synthetic aperture microscopy (ISAM) [31]. ISAM uses a convex lens and a synthetic aperture algorithm to obtain a high resolution in all planes, which is equivalent to the resolution at the focal plane. However, ISAM still requires the mechanical motion of the target or the galvo-scanner. Instead, SVA fully exploits the metasurface, resulting in a compact system and the potential for real-time imaging.

The ability of SVA to significantly increase the DOF and reduce the required resources is highly useful for coherent imaging system with a single transceiver across the electromagnetic spectrum. For the single channel system using dynamic beamforming and scanning, there is always a tradeoff between the DOF and the resolution. The key point is to create a virtual aperture to bridge the connection between the synthetic aperture algorithm and the scanning mechanisms, which facilitates a large DOF reconstruction. Although we demonstrate this technique in a millimeter-wave band with a metasurface, it is potentially more broadly transformative. For example, in terahertz band, quasi-optical systems [32,33] usually require a fixed distance for the best focus quality. Following the idea of SVA, these systems can obtain an extended DOF since the optical focal plane is naturally a virtual aperture.

## 5. Conclusions

In conclusion, we propose a metasurface-based SVA imaging system to produce a 3D high-resolution reconstruction without complex implementations, which avoids stochastic modulation, time-consuming measurement and burdensome computation. SVA first uses a focused beam to obtain a scanning image at the focal plane, meanwhile forming a virtual aperture. Next, an adjusted RMA is applied on the virtual aperture to refocus the targets at all distances. Through the composition of the scanning result and reconstruction result, SVA obtains a high-resolution 3D reconstruction for the whole scene. SVA opens up a new horizon for metasurface-based imaging using simple modulation and digital manipulation, paving the way to practical applications of metasurface-based sensing. Furthermore, it has the potential to significantly improve other single-transceiver beam-scanning coherent imaging setups, which have a very limited DOF. As a proof of principle, our prototype system uses only a small-sized metasurface without optimizing the imaging speed. In the future, we will increase the scale and speed of the system to achieve a higher resolution and a larger FOV in real-time for practical applications.

## Figures and Tables

**Figure 2:**
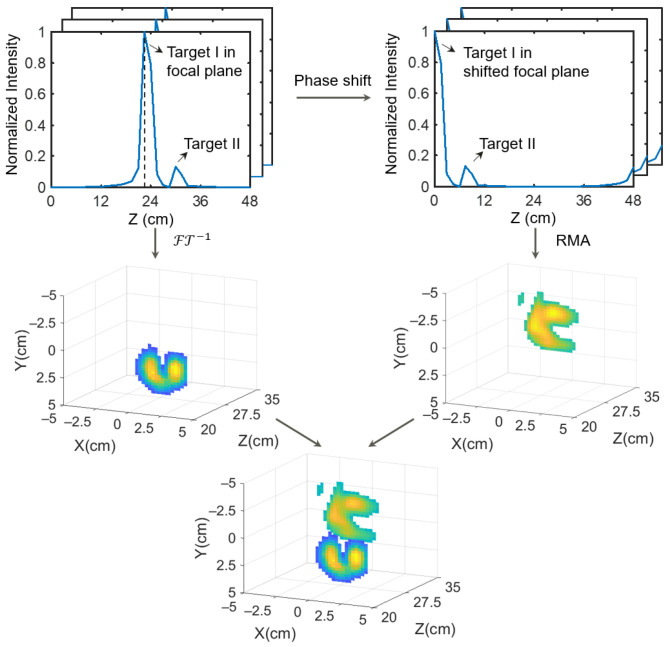
Data processing scheme of the proposed system.

**Table 1:** Comparison of NMSE.

	NBF	SVA	NBF	SVA
	(z = 30 cm)		(z = 40 cm)	
**letter “T”**	0.455	0.181	0.362	0.187
**letter “0”**	0.268	0.059	0.396	0.188
**Two batteries**	0.199	0.096	0.469	0.180

**Table 2:** Comparison of the computation time (s) with that of previous methods.

	SVA	[21]	[24]
**Pre-processing**	0.001	0.25	3.97
**RMA**	0.3	0.01	11.77
**Ratio**	0.003	25	0.337

## Data Availability

The data that support the findings of this study are available from the corresponding author upon reasonable request.

## References

[1] Li L., Zhao H., Liu C., Li L., Cui T.J. (2022). Intelligent metasurfaces: Control, communication and computing. Elight.

[2] Gollub J., Yurduseven O., Trofatter K.P., Arnitz D., Imani M.F., Sleasman T., Boyarsky M., Rose A., Pedross-Engel A., Odabasi H. (2017). Large metasurface aperture for millimeter wave computational imaging at the human-scale. Sci. Rep..

[3] Yurduseven O., Fromenteze T., Decroze C., Fusco V.F. (2020). Frequency-diverse computational automotive radar technique for debris detection. IEEE Sens. J..

[4] Li L., Shuang Y., Ma Q., Li H., Zhao H., Wei M., Liu C., Hao C., Qiu C.-W., Cui T.J. (2019). Intelligent metasurface imager and recognizer. Light Sci. Appl..

[5] Shao Y., Su Z., He H., Jing X., Liu Y., Geng G., Li J., Wang Y., Huang L. (2024). Multispectral imaging through metasurface with quasi-bound states in the continuum. Opt. Express.

[6] Cieślak M.J., Gamage K.A., Glover R. (2016). Coded-aperture imaging systems: Past, present and future development—A review. Radiat. Meas..

[7] Chan W.L., Charan K., Takhar D., Kelly K.F., Baraniuk R.G., Mittleman D.M. (2008). A single-pixel terahertz imaging system based on compressed sensing. Appl. Phys. Lett..

[8] Shen H., Gan L., Newman N., Dong Y., Li C., Huang Y., Shen Y. (2012). Spinning disk for compressive imaging. Opt. Lett..

[9] Hunt J., Driscoll T., Mrozack A., Lipworth G., Reynolds M., Brady D., Smith D.R. (2013). Metamaterial apertures for computational imaging. Science.

[10] Hunt J., Gollub J., Driscoll T., Lipworth G., Mrozack A., Reynolds M.S., Brady D.J., Smith D.R. (2014). Metamaterial microwave holographic imaging system. JOSA A.

[11] Imani M.F., Sleasman T., Smith D.R. (2018). Two-dimensional dynamic metasurface apertures for computational microwave imaging. IEEE Antennas Wirel. Propag. Lett..

[12] Lan G., Imani M.F., Liu Z., Manjarrés J., Hu W., Lan A.S., Smith D.R., Gorlatova M. (2021). MetaSense: Boosting RF sensing accuracy using dynamic metasurface antenna. IEEE Internet Things J..

[13] Sleasman T., Boyarsky M., Pulido-Mancera L., Fromenteze T., Imani M.F., Reynolds M.S., Smith D.R. (2017). Experimental synthetic aperture radar with dynamic metasurfaces. IEEE Trans. Antennas Propag..

[14] Zhao M., Zhu S., Huang H., Hu D., Chen X., Chen J., Zhang A. (2021). Frequency–Polarization Sensitive Metasurface Antenna for Coincidence Imaging. IEEE Antennas Wirel. Propag. Lett..

[15] Zhu S., Dong X., He Y., Zhao M., Dong G., Chen X., Zhang A. (2017). Frequency-polarization-diverse aperture for coincidence imaging. IEEE Microw. Wirel. Compon. Lett..

[16] Watts C.M., Shrekenhamer D., Montoya J., Lipworth G., Hunt J., Sleasman T., Krishna S., Smith D.R., Padilla W.J. (2014). Terahertz compressive imaging with metamaterial spatial light modulators. Nat. Photonics.

[17] Stantchev R.I., Sun B., Hornett S.M., Hobson P.A., Gibson G.M., Padgett M.J., Hendry E. (2016). Noninvasive, near-field terahertz imaging of hidden objects using a single-pixel detector. Sci. Adv..

[18] Stantchev R.I., Yu X., Blu T., Pickwell-MacPherson E. (2020). Real-time terahertz imaging with a single-pixel detector. Nat. Commun..

[19] She R., Liu W., Lu Y., Zhou Z., Li G. (2019). Fourier single-pixel imaging in the terahertz regime. Appl. Phys. Lett..

[20] He Y., Zhang D., Chen Y. (2022). High-resolution wifi imaging with reconfigurable intelligent surfaces. IEEE Internet Things J..

[21] Han J., Li L., Tian S., Liu G., Liu H., Shi Y. (2020). Millimeter-wave imaging using 1-bit programmable metasurface: Simulation model, design, and experiment. IEEE J. Emerg. Sel. Top. Circuits Syst..

[22] Datta S., Tamburrino A., Udpa L. (2022). Gradient index metasurface lens for microwave imaging. Sensors.

[23] Pulido-Mancera L., Fromenteze T., Sleasman T., Boyarsky M., Imani M.F., Reynolds M., Smith D. (2016). Application of range migration algorithms to imaging with a dynamic metasurface antenna. JOSA B.

[24] Molaei A.M., Fromenteze T., Skouroliakou V., Hoang T.V., Kumar R., Fusco V., Yurduseven O. (2022). Development of fast Fourier-compatible image reconstruction for 3D near-field bistatic microwave imaging with dynamic metasurface antennas. IEEE Trans. Veh. Technol..

[25] Zhang J., Hu T., Shao X., Gu H., Xiao Z. (2023). Wavenumber Spectrum Reconstruction Method for Microwave Computational Imaging with Re-Programmable Metasurface. IEEE Trans. Comput. Imaging.

[26] Molaei A.M., Fromenteze T., Hu S., Fusco V., Yurduseven O. (2022). Fourier-based near-field three-dimensional image reconstruction in a multistatic imaging structure using dynamic metasurface antennas. IEEE Trans. Comput. Imaging.

[27] Skouroliakou V., Molaei A.M., Yurduseven O. Towards real-time three-dimensional (3d) imaging using dynamic metasurface antennas. Proceedings of the 2023 17th European Conference on Antennas and Propagation (EuCAP).

[28] Sheen D.M., McMakin D.L., Hall T.E. (2001). Three-dimensional millimeter-wave imaging for concealed weapon detection. IEEE Trans. Microw. Theory Tech..

[29] Ou Z., Wu J., Geng H., Deng X., Zheng X. (2020). Confocal terahertz SAR imaging of hidden objects through rough-surface scattering. Opt. Express.

[30] Goodman J.W. (2005). Introduction to Fourier Optics.

[31] Ralston T.S., Marks D.L., Scott Carney P., Boppart S.A. (2007). Interferometric synthetic aperture microscopy. Nat. Phys..

[32] Cooper K.B., Dengler R.J., Llombart N., Bryllert T., Chattopadhyay G., Schlecht E., Gill J., Lee C., Skalare A., Mehdi I. (2008). Penetrating 3-D imaging at 4-and 25-m range using a submillimeter-wave radar. IEEE Trans. Microw. Theory Tech..

[33] Grajal J., Badolato A., Rubio-Cidre G., Ubeda-Medina L., Mencia-Oliva B., Garcia-Pino A., Gonzalez-Valdes B., Rubinos O. (2015). 3-D high-resolution imaging radar at 300 GHz with enhanced FoV. IEEE Trans. Microw. Theory Tech..

